# Chemical and technological attributes of sugarcane as functions of organomineral fertilizer based on filter cake or sewage sludge as organic matter sources

**DOI:** 10.1371/journal.pone.0236852

**Published:** 2021-12-15

**Authors:** Carlos André Gonçalves, Reginaldo de Camargo, Robson Thiago Xavier de Sousa, Narcisa Silva Soares, Roberta Camargos de Oliveira, Mayara Cristina Stanger, Regina Maria Quintão Lana, Ernane Miranda Lemes

**Affiliations:** 1 Lutheran University of Brazil (ULBRA), Agronomy, Campus Itumbiara, Itumbiara, GO, Brazil; 2 Science Agrarian Institute (ICIAG), Federal University of Uberlândia (UFU), Campus Glória, Uberlândia, MG, Brazil; 3 Vale do Tijuco Sugarcane Mill, Sugar and Alcohol Company of Minas Gerais State (CMAA), Uberaba, MG, Brazil; Kyonggi University, REPUBLIC OF KOREA

## Abstract

Sugarcane is one of the main alternative sources of biomass for the biofuel sector, and its large-scale production has considerable environmental impact. Organomineral fertilizers formulated with potential environmental contaminants, such as filter cake and sewage sludge, positively influence plant growth and development. The objective of the present study was to evaluate the chemical and physical characteristics of sugarcane fertilized with pelletized organomineral fertilizers based on filter cake or sewage sludge. Eight field treatments were applied, based on three organomineral fertilizer compositions (50%, 100%, and 150%) associated with two organic matter (OM) sources (filter cake or sewage sludge), in addition to a control with 100% mineral fertilizer application, and a no-fertilization control (0%). Sugarcane attributes were evaluated during two consecutive harvests. The weights of stalks per hectare (ton ha^-1^), sugarcane productivity (ton ha^-1^), quantity of sugar per hectare (TSH, ton ha^-1^), and physicochemical properties of sugarcane juice (pol [%], Brix [%], purity [%], and fiber [%]) were evaluated. There were no significant differences in the attributes between OM sources or organomineral fertilization treatments and the exclusive mineral fertilization. The organomineral fertilizer application rate recommended for maximum quantitative and qualitative sugarcane in the first sugarcane harvest was between 2 and 9% above the regular recommendation for mineral fertilizer, regardless of the OM source. In the second harvest, the sewage sludge source increased total sugar and sugarcane per hectare by 4.68 and 4.19%, respectively, compared to the sugarcane filter cake source. Sewage sludge and sugarcane filter cake are viable alternatives for organomineral composition and could improve economic returns and minimize negative environmental impacts in sugarcane cultivation systems.

## Introduction

The replacement of fossil fuels with renewable, economically viable, and less polluting sources has increasingly been demanded by stakeholders and has become feasible over the past few decades. Ethanol, mainly obtained from sugarcane and maize, is an example of a substitute that has been adopted on large scales. Brazil and the United States have begun investing heavily in such biofuels. The United States has focused on ethanol from maize and is the largest producer, with more than 60 billion liters produced per year [1]. In 2020/21, Brazil achieved a record production of 29.74 billion liters of ethanol (anhydrous and hydrated) from sugarcane; maize was responsible for the generation of only 3.02 billion liters [2].

Sugarcane is considered one of the major alternative sources of biomass for the biofuel sector because of its high potential for ethanol production and associated by-products. Sugarcane mills have pursued efficiency in ethanol production, leading to an increase in its supply while reducing its production costs. Concomitantly, the ethanol industry seeks the use sustainable agricultural inputs and eco-friendly cultivation techniques. Organomineral fertilizers are an excellent example of such improved technologies and environmental payback.

Organomineral fertilizers are mixtures of mineral fertilizers of natural or synthetic origin and organic compounds of natural origin or derived from industrial processes–agro and rural or urban by-products. Such fertilizers can be fluid or solid and can be applied via soil, leaf, fertigation, hydroponics, and seeds [3]. The remarkable efficiency of such fertilizers in many crops has gradually been acknowledged in the market. Additionally, organominerals use environmental liabilities that can contaminate the environment when they are not deposited in appropriate destinations.

Understanding sugarcane nutritional requirements is essential for the study of crop fertilization (organic, mineral, or organominerals) based on the precise amounts of nutrients required [4]. However, the amounts of nutrients extracted by plants are variable and depend on the crop variety, cycle, soil management, and nutrient availability. The evaluation of eight sugarcane cultivars in the “Z*ona da Mata*” biome (forest area) in Minas Gerais state (Brazil) revealed that the accumulation of nitrogen (N), phosphorus (P), potassium (K), calcium (Ca), and magnesium (Mg) in the plant shoots ranged from 114.8 to 160.5, 38.3 to 42.7, 206.5 to 288.4, 109.8 to 161.8, and 19.5 to 39.5 kg ha^-1^, respectively; on average, the nutrient extraction order was K > N >Ca > P >Mg [5].

Another study in the same biome, but in Pernambuco state (Brazil), reported that the average accumulations of N, P, K, Ca, and Mg nutrients by sugarcane were 179, 25, 325, 226, and 87 kg ha^-1^, respectively, with an extraction order of K > Ca > N > Mg >P [6]. Such findings from different studies illustrate the importance of constantly studying plant nutrient dynamics to facilitate precise fertilization.

From July 23, 2009, organomineral fertilizers in Brazil have to satisfy the Normative Instruction No. 25 regulation of the Brazilian Ministry of Agriculture, Livestock and Food Supply [7], which requires that organic matter (OM) used has a minimum organic carbon content of 8% and corresponds to approximately 40% of the total OM and 60% of mineral fertilizer. More nutrients can be declared on the label and presented based on soluble and total levels.

Different sources of OM are used in organomineral fertilizer development, including industrial filter cakes, which are extensively available and exhibit low variation in chemical composition. According to the Union of Sugarcane Industries [8], Brazil harvest in 2020/21 registered approximately 600 million tons of processed sugarcane material in a cropped area of more than 10 million ha. Such figures highlight great potential for sugarcane filter cake production.

Filter cake comprises a mixture of ground bagasse and decanting sludge derived following sugarcane juice treatment. Filter cake emerges from the rotary filters after the extraction of residual sucrose from the sludge [9]. A survey conducted by the National Bank for Economic and Social Development (BNDES) [10], based on data from the 2015/2016 sugarcane harvest in Brazil, indicated that for each ton (1,000 kg) of processed sugarcane, approximately 100–400 kg of filter cake was generated, yielding 66.6–266.2 million tons of the residue annually.

Sugarcane filter cake is the main organic residue in the organomineral fertilizer. Such cakes were initially obtained from sugar production processes; however, currently, alcohol mills are also producing considerable amounts [11]. Sugarcane productivity is reportedly enhanced when filter cake is applied to the soil (directly or as an organomineral) [11–13]. In addition, sugarcane filter cake consists of about 70% moisture, Ca, K, Mg, and N, and about 1.2 to 1.8% P and has high OM content. Due to such characteristics, the use of filter cake as a component in organomineral fertilizer has been on the rise since 1999, when the prices of mineral fertilizers reached high levels. In addition, consumer environmental awareness has increased [14].

The evaluation of the quality and productivity of sugarcane indicated that the use of filter cake enriched with soluble phosphate and applied in furrows at planting could partially replace mineral phosphate fertilizer [15]. Organomineral fertilizers formulated with filter cake and treated sewage sludge enhance the growth and development of soybean plants and improve the activity of enzymes associated with the protection of cell membranes [16]. The term biosolid is reserved for stabilized by-products, such as sewage sludge; otherwise, the terms cake, sludge, or solid are used [17, 18]. The use of sewage sludge in agriculture was initiated formally in Brazil by the CONAMA Resolution No. 375/2006 [19], which established criteria for the safe use of the residue as fertilizer.

The benefits of the use of organomineral fertilizers formulated from different sources of OM have been observed in several studies, including in crops, such as sugarcane [20], soybean [21], corn [22], onion [23], beans [24], tomato [25], and potatoes [26]. Filter cakes are consolidated organic sources of organominerals [15, 27], mainly because of their high availability; treated sewage sludge has also been studied extensively as a potential crop fertilizer. However, only recently has sewage sludge been tested as a potential component of organomineral fertilizer [28].

Incorporating sewage sludge into the soil can improve its fertility and reduce the potential acidity in proportion to the dose of fertilizer applied [29]. Such a property is attributable the use of lime in the treatment of sewage sludge [30]. However, due to improvements in logistical and technological factors associated with crop cultivation activities, sources of OM, such as filter cake and treated sewage sludge, have begun to exhibit commercial viability, especially when mixed with mineral fertilizers to establish organomineral fertilizers.

Organomineral fertilizers have distinct characteristics; for example, P derived from such fertilizers is more rapidly available than P derived from mineral sources (triple superphosphate) [31]. Organomineral fertilizers also offer higher exchangeable K contents in the upper soil layers and are relatively efficient compared to exclusive mineral fertilizer applications. Organomineral fertilizers could enhance sugarcane stalk production by 15% when compared to mineral fertilizers [32].

High sugarcane productivity must be accompanied by appropriate physicochemical properties to ensure high sugar and alcohol yields. The yield and productivity of sugarcane and other crops are influenced by the quantity and balance of nutrients in the soil. Sugarcane productivity, plant height, and stem diameter are positively correlated with organomineral fertilizer application rates at planting [33]. In sorghum, a plant in the Poaceae family and morphologically and physiologically similar to sugarcane, organomineral fertilizer supplementation influences ethanol, reducing sugars, and total recoverable sugars production [34].

The use of sewage sludge has already been demonstrated to be feasible from a sanitary point of view [30], through disinfection at high efficiency and from an agronomic perspective for the fertilization of sugarcane [33]. Sewage sludge-based fertilizers supplemented with mineral fertilizers are viable alternatives and improve agronomic parameters; however, an evaluation of their effects on industrial sugarcane yield parameters is required. Consequently, the objective of the present study was to evaluate the physicochemical quality of sugarcane fertilized with pelletized organomineral fertilizers based on filter cake and sanitized sewage sludge, in two consecutive sugarcane crop cycles.

## Material and methods

### Experimental area

The study was conducted at an experimental area of the *Companhia Mineira de Açúcar e Álcool* (CMAA), *Vale do Tijuco* Unity (*São José* experimental farm), located in Prata municipality, Minas Gerais state, Brazil, at 19° 29’ 59” S and 48° 28’ 26” W and 780 m above sea level. The climate of the experimental area is tropical, semi-humid and classified as Aw, tropical dry winter, according to Köppen [35]. Rains are concentrated between November and March, with an average annual rainfall of 1,400 mm, and the drought period is concentrated between July and August–September. The average maximum temperature occurs from November to February, ranging from 31°C to 36°C [36].

The experiment was implemented upstream of a hill in a soil classified as dystrophic yellow latosol (Oxisol) [37]. The soil was characterized as sandy soil, with 72% sand, 18.5% clay, and 9.5% silt. Soil sampling was performed at 0–0.2 and 0.2–0.4 m depths and their chemical analyses are described in Table 1.

**Table 1:** Soil physicochemical properties in the experimental area at 0–0.2 and 0.2–0.4 m depths.

Depth (m)	pH (H_2_O)	Ca	Mg	Al	P	K	H+Al	CEC	V	m	O.M.
		--cmol_*c*_ dm^-3^--		--mg dm^-3^--		-cmol_*c*_ dm^-3^-		-----%-----		---g kg^-1^---	
0–0.2	5.7	1.1	0.5	0	6.7	88	1.2	3.03	60	0	2.0
0.2–0.4	4.7	1.0	0.3	0.2	2.3	70	1.6	3.08	48	9.0	1.4

Depth (m) = soil depth; pH in water (1:2, 5); Ca, Mg, Al = KCl 1 mol L^-1^; Available P = Mehlich^-1^; K = HCl 0.05 mol L^-1^ + H_2_SO_4_ 0.0125 mol L^-1^; H + Al = SMP buffer solution at pH 7.5; CEC = cation exchange capacity at pH 7; V = base saturation; m = aluminum saturation, O.M. = soil organic matter, colorimetric method. Methodologies were based on [38].

The soil in the experimental area received 2.4 t ha^-1^ of dolomitic lime, 1.5 t ha^-1^ of gypsum, and 100 kg ha^-1^ of P_2_O_5_. A moldboard plow (cutting width of 0.29 m with 0.81 m spacing) and leveling harrow (disks of 0.91 × 0.56 m) were used in the area. A degraded pasture has occupied the experimental site over the last 10 years.

### Organomineral fertilizers

At planting, 570 kg ha^-1^ of the mineral formulation 21-04-07 (N, P_2_O_5_, and K_2_O), and 570 kg ha^-1^ of the mineral formulation 07-00-28 (N, P_2_O_5_, K_2_O) + 0.7% boron were applied 150 days after planting [39]. Both formulations of organomineral fertilizer were produced using either treated sewage sludge (biosolids) or sugarcane filter cake sources of organic matter.

**Fig 1:**
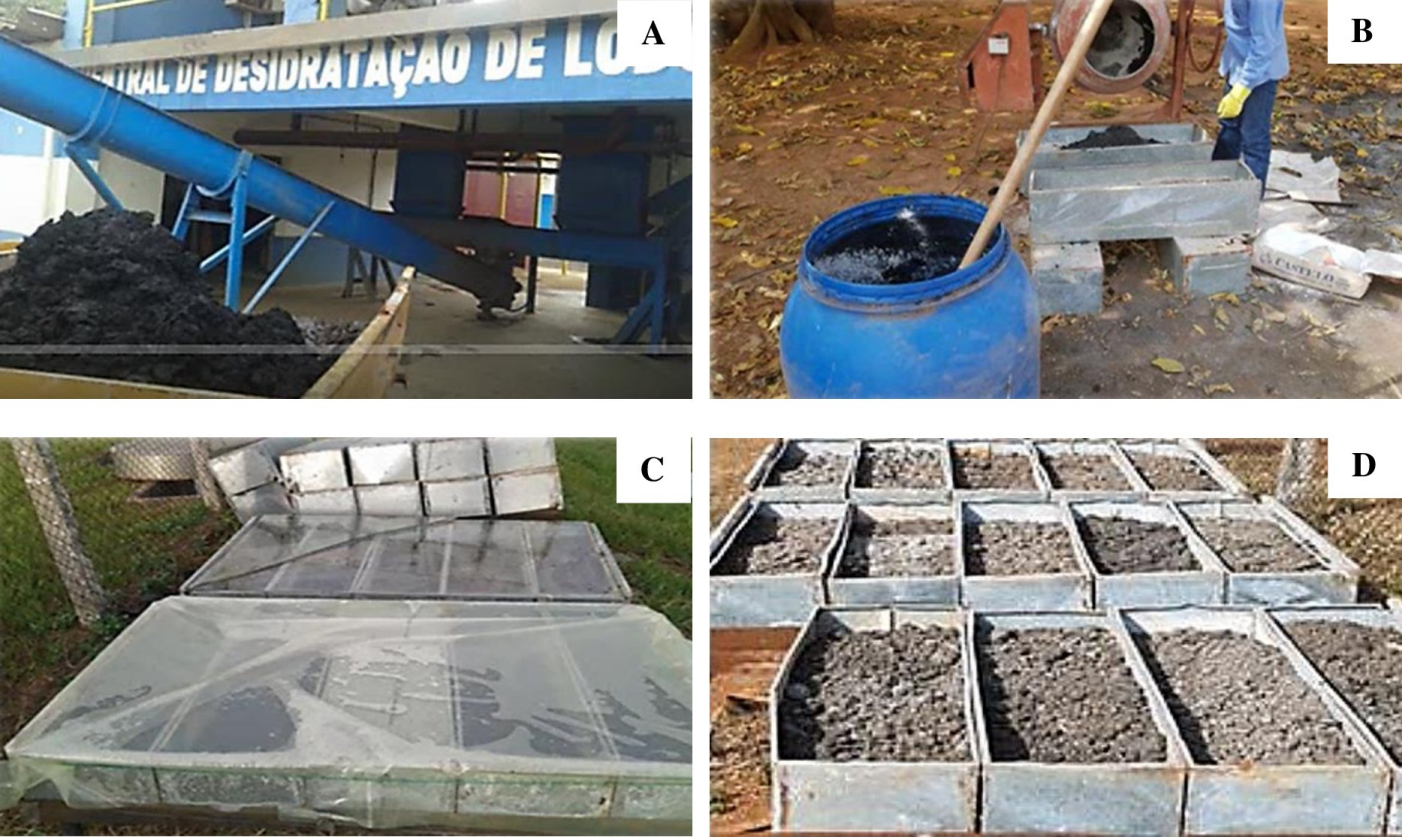
Process of sewage sludge treatment. A. solid residue after centrifugation; B. treatment with hydrated lime; C. exposure to sunlight; D. drying in the sun.

Treated sewage sludge was collected from the Municipal Department of Water and Sewage Treatment in Uberlandia, Brazil. The sewage sludge treatment began with centrifugation to separate the solids and reduce moisture to 70%. The wet sewage sludge was subjected to chemical treatment by incorporating 30% hydrated lime [Ca(OH)_2_], and then packed in rectangular boxes of galvanized zinc (0.3 × 0.3 × 1 m). The structure was covered with a transparent canvas and exposed to sunlight for 15 days. Subsequently, the canvas was removed, the mixture left to dry for 30 days, and stabilized at approximately 20% moisture (Fig 1).

According to the laboratorial report and the fertilization need for sugarcane planting, a pelletized organomineral was prepared and composed of 39.3% biosolid, 12.2% crumbled KCl (58% K_2_O), 47% crumbled monoammonium phosphate (12% N and 44% P_2_O_5_), and 1.5% water. The side dressing fertilization (broadcast fertilizer application) received organomineral fertilizer composed of 31% biosolid, 15% polymerized urea (45% N), 48.3% crumbled KCl (58% K_2_O), 4.2% boric acid, and 1.5% water.

Pelletized organomineral fertilizer based on sugarcane filter cake was produced by Geociclo Biotechnology S/A (Uberlândia, Brazil). The composted filter cake residues were composed of soluble mineral macronutrients (urea, monoammonium phosphate, and KCl), micronutrients (oxisulfates), and boron as ulexite. The resulting product received an organic polymer to produce organomineral pellets. The organomineral pellets were approximately 3.9 mm in diameter and 9.1 mm in length.

The chemical characteristics of the sewage sludge and sugarcane filter cake used are listed in Table 2. The sewage sludge chemical characteristics satisfied the standards prescribed by CONAMA Resolution No. 375/2006 [19], and the values were close to those reported by Moraes et al. [33].

**Table 2:** Chemical characteristics of sewage sludge and sugarcane filter cake used to establish the organomineral fertilizer.

Attribute	Unity	Sewage sludge	Filter cake	Attribute	Unity	Sewage sludge	Filter cake
pH CaCl_2_	pH	8.1	5.9	Total mineral	%	51	32
Density	g cm^-3^	0.66	0.65	Boron	mg kg^-1^	10	< LQ
Total N	%	0.99	1.79	Sodium	mg kg^-1^	201	-
O.M. Total	%	50	35	Manganese	mg kg^-1^	209	460
Total carbon	%	28	10	Copper	mg kg^-1^	135	< LQ
C/N relation		28/1	20/1	Zinc	mg kg^-1^	1042	< LQ
Phosphorus	%	2.80	2.25	Iron	mg kg^-1^	27,236	11,980
Potassium	%	0.30	0.30	Cadmium	mg kg^-1^	1.4	2.1
Calcium	%	8.25	2.43	Mercury	mg kg^-1^	0.7	< LQ
Magnesium	%	2.48	0.26	Chrome	mg kg^-1^	931	94.2
Sulfur	%	1.31	0.39	Nickel	mg kg^-1^	250	-

N—[Total N] = sulfuric digestion; P, K, Ca, Mg, S, Cu, Fe, Mn, and Zn = nitro perchloric digestion; B = colorimetric azomethine H; O.M. = organic matter; < LQ: lower than limit of quantification. Methodologies were based on [40].

The Geociclo Biotechnology S/A (Uberlândia, Brazil) also performed microbiological (total and thermotolerant coliforms) and chemical analyses of the organominerals. The total concentrations of heavy metals (cadmium [Cd], chromium [Cr], nickel [Ni], and lead [Pb]) and the levels of total and thermotolerant coliforms were within the acceptable values prescribed by CONAMA Resolution No. 375/2006 [19]; therefore, it was considered acceptable for use as an agricultural fertilizer.

### Experimental design

The experiment was carried out based on a completely randomized design in a 3×2+2 factorial scheme (eight treatments), with three application rates of pelletized organomineral fertilizer (50%, 100%, and 150% of the recommended mineral fertilizer dose), two organic sources (sewage sludge and filter cake), and two controls (positive control: exclusive mineral fertilization and negative control: no fertilization) (Table 3), with four replicates.

**Table 3:** Soil fertilizer treatments applied to sugarcane crop.

Treatment	Application Rate (kg ha^-1^)
Organomineral Fertilizer–sewage sludge base 50%	285
Organomineral Fertilizer–sewage sludge base 100%	570
Organomineral Fertilizer–sewage sludge base 150%	855
Organomineral Fertilizer–filter cake base 50%	285
Organomineral Fertilizer–filter cake base 100%	570
Organomineral Fertilizer–filter cake base 150%	855
Exclusive Mineral fertilizer–positive control	570
No fertilizer application–negative control	0

The experimental plots were 10 m long and 6 m wide (60 m^2^) and included five sugarcane planting lines (1.5-m spacing between lines). A 3-m gap separated each plot from the adjacent one. The experimental area in the plots included three planting lines at the center of each plot.

**Fig 2:**
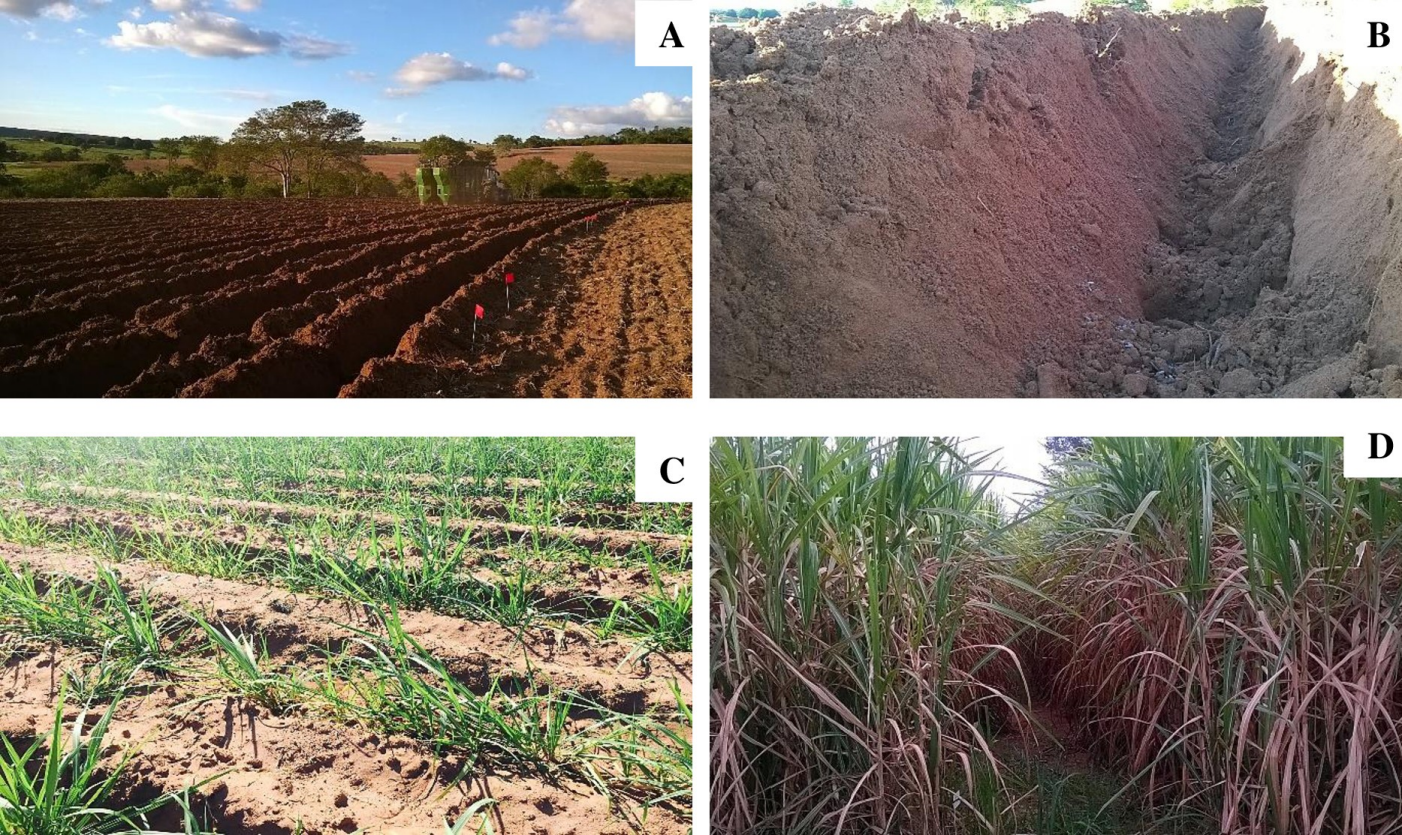
A) Overview of the experimental area after the establishment of the planting grooves; B) planting grooves in detail; C) Experimental set at four months after planting; D) Experimental set up at 12 months after planting.

The sugarcane variety used in the present study was “RB 92579,” which is a genotype with a slightly decumbent growth habit, biometric uniformity (stalk diameter and plant height), and uniform tillering. The genotype originated from a selection of genotypes from the Northeast region of Brazil and is characterized by long-lived sugarcane ratoons with high productivity, good closing of the between-line, and efficient planting and harvesting [41]. Sugarcane planting was mechanized and was performed in the last week of May (Fig 2). In the planting furrows, 15 to 18 viable buds were sown per linear meter at a depth of 0.3 to 0.4 m. Organomineral fertilizers were applied during planting.

Sugarcane quality was evaluated during two consecutive cuts (cycles): sugarcane in the first year (sugarcane plant) and sugarcane in the second year (first year of ratoon sugarcane). Insect pest (ants and termites) control was performed with the application of 2.5 g ha^-1^ of fipronil. Weed infestation was managed with the herbicides hexazinone, diuron, and monosodium methanearsonate (MSMA) at doses of 5, 3.2, and 3 L ha^-1^, respectively.

### Technological and chemical analyses

Ten representative sugarcane stalks were sampled from the three central planting lines for physicochemical analyses. The stalks were clipped at the apical bud (breakpoint) and transported to the CMAA Laboratory for analyses. Sample collections occurred 366 days after planting and 376 days after the first cut.

The estimated weight of stalks per hectare (ton ha^-1^) (StH), sugarcane biomass productivity (ton ha^-1^) (Prod.), and the quantity of sugar per hectare (ton ha^-1^) (TSH) were evaluated. Stalk weight per hectare was estimated by considering average stalk diameter, height, and tillering. Processing was carried out according to a methodology based on sucrose content. After the disintegration and homogenization of the sugarcane stalks, a sample was collected (0.5 kg) and juice extracted using a hydraulic press used for physicochemical analyses [42].

The variables evaluated were as follows: sugarcane pol (%): the quantity of sucrose (in percentage) in the sugarcane juice; Brix (%):the percentage (weight/weight) of soluble solids contained in the juice, i.e. the content of sucrose in the sugarcane juice; purity (%): is based on the “pol/Brix×100” equation (the higher the sugarcane purity, the better the quality of the juice for sugar recovery; and fiber (%):the water-insoluble biomass in the sugarcane.

### Statistical analysis

Extreme values (outliers) in all variables evaluated were identified using boxplot graphs of residuals [43] generated using IBM SPSS® Statistics v. 20 (IBM Corp., Armonk, NY, USA). The outliers identified were calculated as *lost parcels* to replace the extreme values.

IBM SPSS Statistics v. 20 was also used to test for the Analysis of Variance (ANOVA) assumptions: Shapiro-Wilk [44] test for normality and Levene’s [45] test for homogeneity of variance, both at p > 0.01.

After testing for the assumptions, the data were subjected to ANOVA (*F* test) to detect interactions between the factors (organic sources × doses) and differences among levels of each factor (p < 0.05) [46]. Appropriate comparisons were performed following detection of significant differences.

The results of the additional treatments (mineral fertilization and no-fertilization) were compared with the experimental treatments using Dunnett’s test [47] (p < 0.05) using Assistat® v. 7.7 beta.

The means of the experimental treatments were compared using Tukey’s test [48] (organic sources) at p < 0.05, and by regression analysis (organomineral doses) using SISVAR® v. 5.3. The regression model was determined based on the significance of the coefficients (p < 0.05) and the coefficient of determination (R^2^ > 70%). Sigma Plot® v.12 (Systat Software Inc., San Jose, CA) was used to illustrate graphs showing the effects of the various organomineral application rates on sugarcane yield characteristics.

Sugarcane crop yield characteristics between the first (366 days after planting) and second (376 days after the first harvest) sugarcane harvest were compared via joint analyses using Tukey’s test [48].

## Results

The application of organomineral fertilizer based on treated sewage sludge at a dose of 100% (SS100) resulted in higher stalk production per hectare in the first year than in the second year (Table 3).

Lack of mineral fertilization (negative control) resulted in lower sugarcane productivity and production than the positive control at both harvesting cycles. The SS100 treatment reflected a greater quantity of sugar produced per hectare (TSH) in the second cycle, which was 31.5% higher than that obtained in the negative control. In addition, the negative control yielded lower sucrose contents (pol) and soluble solids (Brix) in sugarcane syrup in the first cycle of assessment when compared to the mineral fertilizer treatment (Table 4); however, in the second harvesting cycle, only sugarcane juice purity was lower in the negative control.

**Table 4:** Average chemical, physical, and biometric properties of the RB 92579 sugarcane cultivar (366 days after planting—first cut; 376 days after first harvest—second cut) under fertilization treatments, including organominerals, based on treated sewage sludge (SS) and sugarcane filter cake (Fk).

Treat.	Pol (%)		Brix (%)		Purity (%)		Fiber (%)		StH (ton ha^-1^)		TSH (ton ha^-1^)		Prod (ton ha^-1^)	
	First	Second	First	Second	First	Second	First	Second	First	Second	First	Second	First	Second
SS50	15.35	14.39	20.68	19.19	89.50	89.70	13.45	12.79	74.11	65.59	23.65	21.41	157.34	139.23
SS100	15.72	14.08	20.82	19.43	89.50	90.22	13.59	13.02	100.3*	90.76	24.37	22.61	159.12	139.79
SS150	15.33	14.21	20.81	18.92	88.69	90.35	13.14	13.06	83.34	81.70	23.45	22.26	152.99	140.93
FC50	14.87	14.44	20.48	19.19	88.61	89.81	13.90	12.64	79.15	80.36	24.21	21.19	162.80	143.36
FC100	15.22	13.63	20.61	18.40	89.02	88.79	13.19	12.90	83.05	78.08	23.41	20.48	153.87	135.78
FC150	15.04	14.10	20.59	18.69	88.77	90.14	13.63	12.71	81.65	75.86	23.97	20.84	159.36	139.96
NC	13.88*	12.91	19.15*	17.76	87.70	87.12*	13.05	12.89	72.65	53.86	20.97*	17.95*	151.12*	124.28*
Control	15.03	14.16	20.51	18.94	88.98	90.54	13.59	13.44	76.29	74.61	23.90	21.55	158.91	140.41
LSD	0.77	1.27	0.86	1.43	2.04	3.09	0.63	1.52	18.53	21.78	1.87	9.82	7.75	2.32
CV (%)	2.55	4.58	2.40	3.82	1.14	1.73	2.33	5.86	11.49	14.92	4.01	3.59	2.47	5.58
S.W.	0.10	0.09	0.11	0.10	0.11	0.08	0.14	0.09	0.15	0.13	0.11	0.09	0.10	0.15
L.	0.56	0.26	2.24	0.54	1.47	0.52	1.40	2.10	1.38	0.70	0.66	0.64	1.42	2.25

Treat: fertilization treatments applied to sugarcane crops. SS: organomineral fertilizer based on treated sewage sludge. FC: organomineral fertilizer based on sugarcane filter cake. NC: negative control. Pol: apparent sucrose percentage in sugarcane syrup; Brix: soluble solid percentage in sugarcane syrup; Purity: Pol/Brix×100 (%); Fiber: water-insoluble biomass percentage. StH: sugarcane stalk per hectare (ton ha^-1^); TSH: total sugar produced per hectare (ton ha^-1^); Prod: Sugarcane biomass productivity (ton ha^-1^).

*: average differs from that of the control treatment (mineral fertilization) based the Dunnett’s test (p < 0.05). LSD: least significant difference. S.W.: Shapiro-Wilk test for normality (p > 0.01: normal distribution). L.: Levene’s test for homogeneity of variance (p > 0.01: all are homogenous).

**Fig 3:**
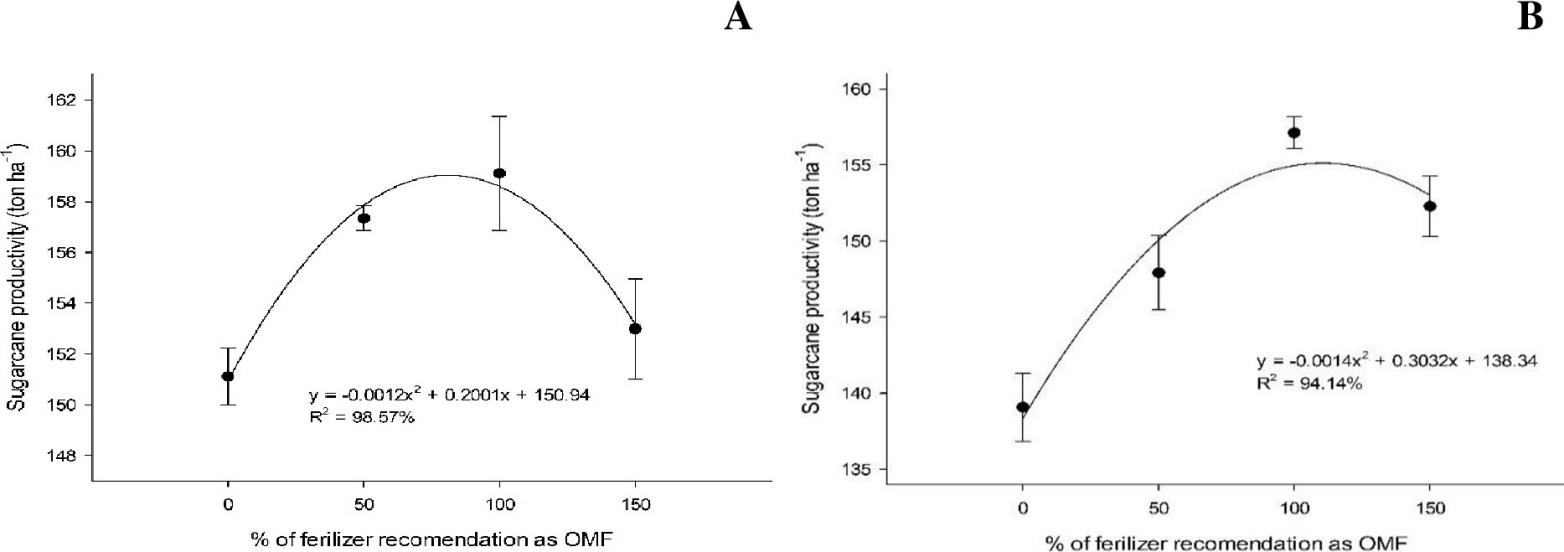
Sugarcane biomass productivity (ton ha^-1^) under organomineral fertilizer amendment based on sewage sludge in the first (A) and second cut (B).

In the first cutting cycle, there was a significant interaction between the OM source used to formulate the organomineral fertilizer and the application rate for sugarcane productivity. Only the sewage sludge source had a significant (p < 0.05) polynomial relationship with yield (Fig 3A), with the highest yield (159.3 ton ha^-1^) obtained under an organomineral fertilizer application rate of 83.4%. The filter cake source, under which no polynomial relationship was observed, yielded a sugarcane productivity of 159.4 ton ha^-1^ under 150% (FC150) organomineral fertilizer.

In the second cutting cycle, there were no interaction effects between sources and doses on sugarcane productivity; therefore, the effect of the dose was independent of the effect of OM source adopted (Fig 3B). The highest sugarcane productivity observed was 154.8 ton ha^-1^, which was obtained with an organomineral fertilizer application rate of 108.3%.

The highest yield at the first cut was observed when 50% organomineral fertilizer, based on sugarcane filter cake (162.8 ton ha^-1^), and was approximately 7.73% higher than that of the negative control (151.1 ton ha^-1^). At a similar dose, the organomineral fertilizer based on sugarcane filter cake resulted in high fiber production in the first cut (13.9%) when compared with the fiber produced under the negative control (13.1%) (Table 4).

**Fig 4:**
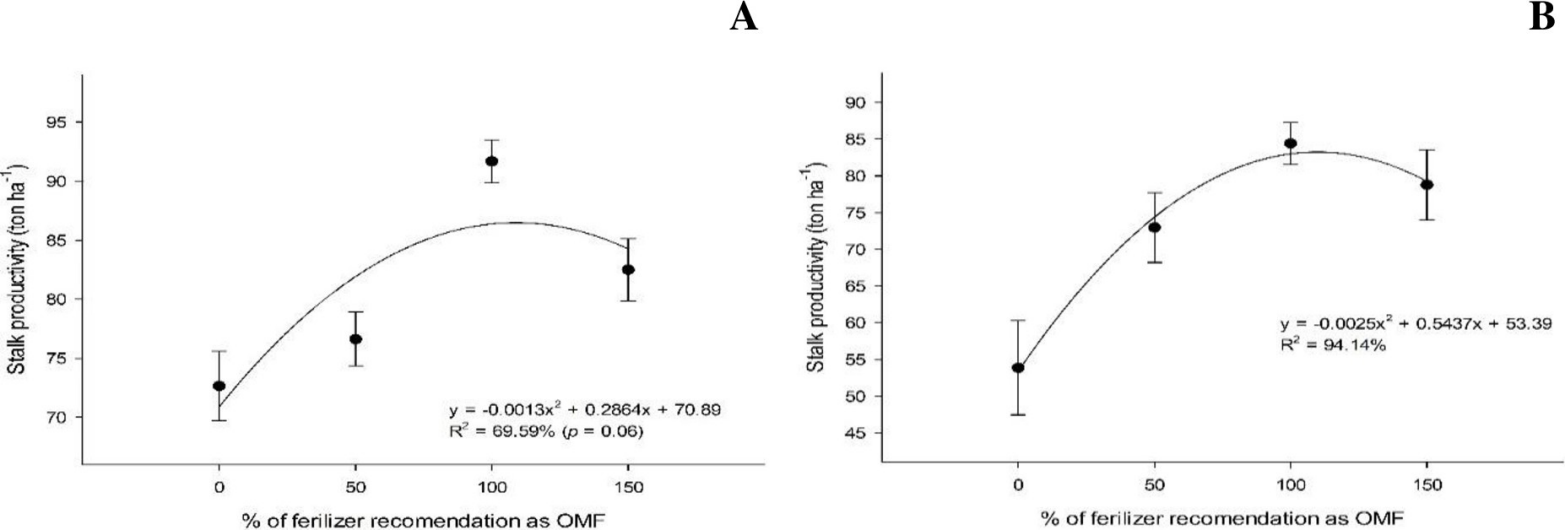
Stalk productivity (ton ha^-1^) under organomineral fertilization at the first (A) and second cut (B).

The highest stalk productivity per hectare at the first cut was 86.7 ton ha^-1^, which was obtained under an organomineral fertilizer application rate of 110.2%, regardless of the source of the OM. In the second cut, the highest stalk productivity was 83.1 ton ha^-1^, which was obtained at a 108.7% organomineral fertilizer application rate (Fig 4). Notably, maximum stalk productivity was generally similar between cuts, with a minor increase in productivity (4.3%) in the first cut.

At the first cut, when the sewage sludge was the basis for the organomineral fertilizer, the sucrose content (pol) was 2.16% higher than that observed when sugarcane filter cake was the basis for the organomineral fertilizer (Table 4). The sewage sludge source increased the total sugar per hectare and sugarcane production by 4.68% and 4.18%, respectively, in the second cut, when compared with the yields under organomineral fertilizer associated with filter cake (Table 5).

**Table 5:** Average sucrose content (pol), sugarcane productivity, and total sugar obtained following fertilization with organomineral fertilizer associated with treated sewage sludge and filter cake.

Source	Pol (%)	TSH (ton ha^-1^)	Prod (ton ha^-1^)
	-----First cut-----	-----------Second cut-----------	
Filter cake	14.75 b^1^	20.11 b	146.04 b
Sewage sludge	15.07 a	21.05 a	152.15 a
LSD	0.28	0.76	4.06
CV (%)	2.62	5.07	3.73
S.W.	0.10	0.09	0.15
L.	0.56	0.64	2.25

^1^Averages followed by distinct letters in a row are significantly different according to Tukey’s test at a 0.05 significance level. S.W. Shapiro-Wilk test for normality. L.: Levene’s test for homogeneity of variance. Pol: sucrose content in sugarcane syrup. TSH: total sugar produced per hectare (ton ha^-1^) Prod: sugarcane biomass productivity (ton ha^-1^).

**Fig 5:**
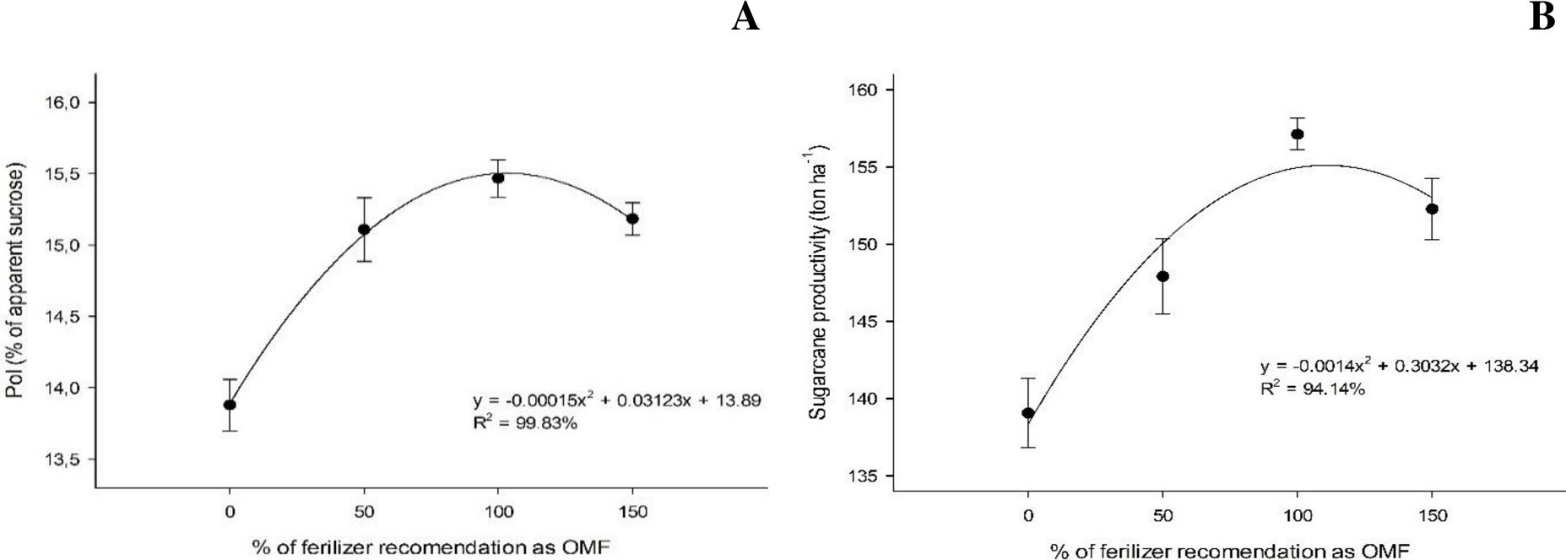
Sugarcane sucrose content (pol, %) under different organomineral fertilizer application rates at the first (A) and second cut (B).

The highest sucrose contents (pol) observed at the first and second cuts were 15.5% and 14.3%, respectively, under 104.1 and 102.1% organomineral fertilizer application rates, regardless of the sources of OM adopted (Fig 5).

**Fig 6:**
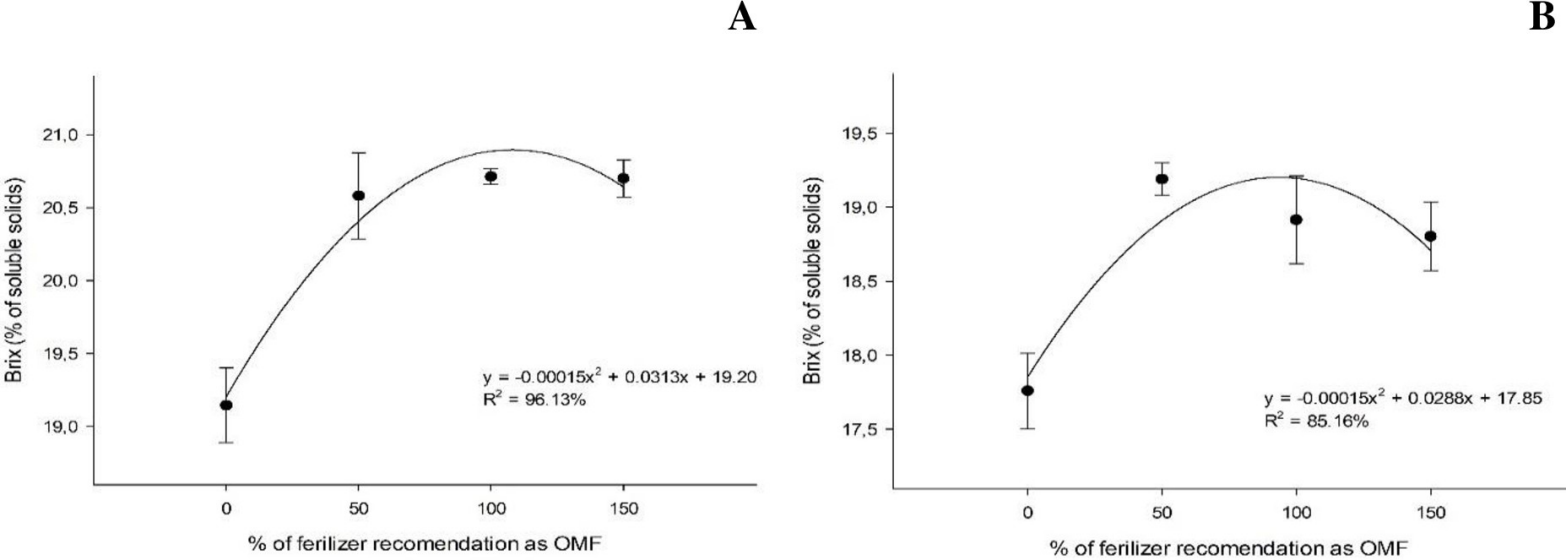
Percentages of soluble solids (Brix, %) under organomineral fertilizer treatments at the first (A) and second cut (B).

The percentages of soluble solids (Brix) varied considerably under different organomineral fertilizer application rates in the two harvest cycles, independent of the source of OM adopted. The highest percentages of soluble solids at the first and second cuts were 20.8% and 19.2%, respectively, under 104.3 and 96% organomineral application rates (Fig 6).

**Fig 7:**
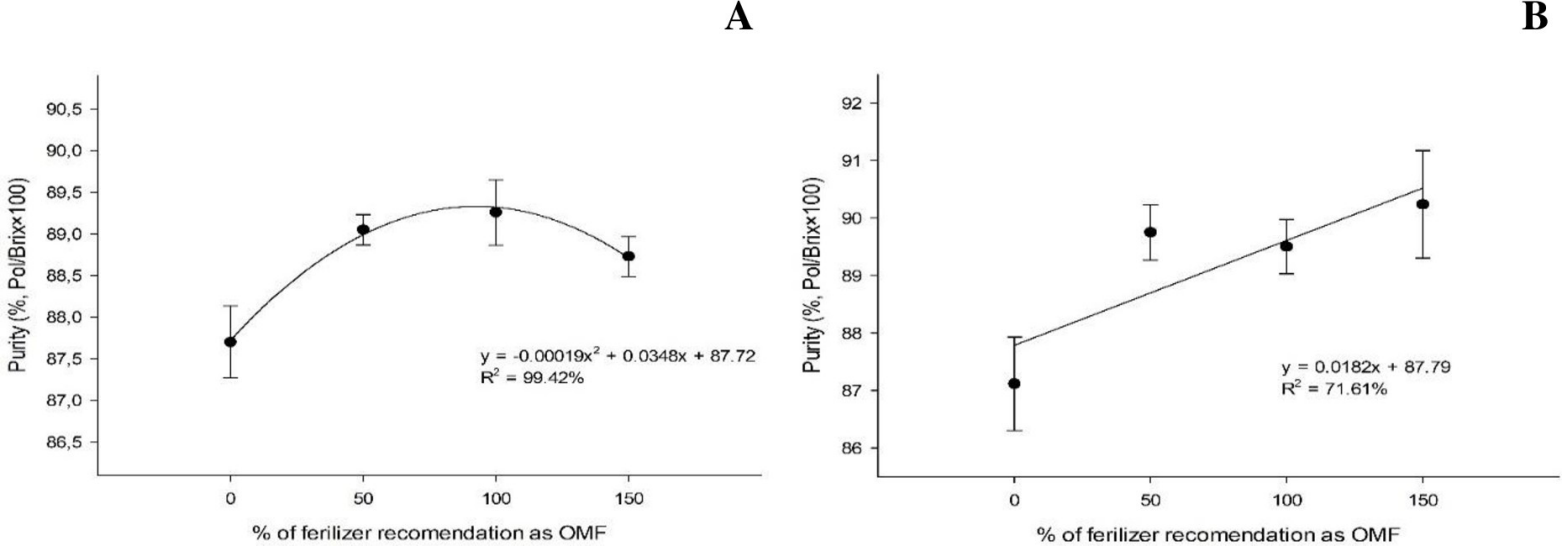
Sugarcane juice purity (%) under organomineral fertilizer application at the first (A) and second cut (B).

At the first cut, the highest sugarcane juice purity (89.3%) was observed under an organomineral fertilizer application rate of 91.6%, using either sewage sludge or sugarcane filter cake as the source of OM. At the second cut, for each kilogram of organomineral fertilizer applied, using sewage sludge or sugarcane filter cake, there was a 0.0182% increase in the purity of the sugarcane juice (Fig 7).

**Fig 8:**
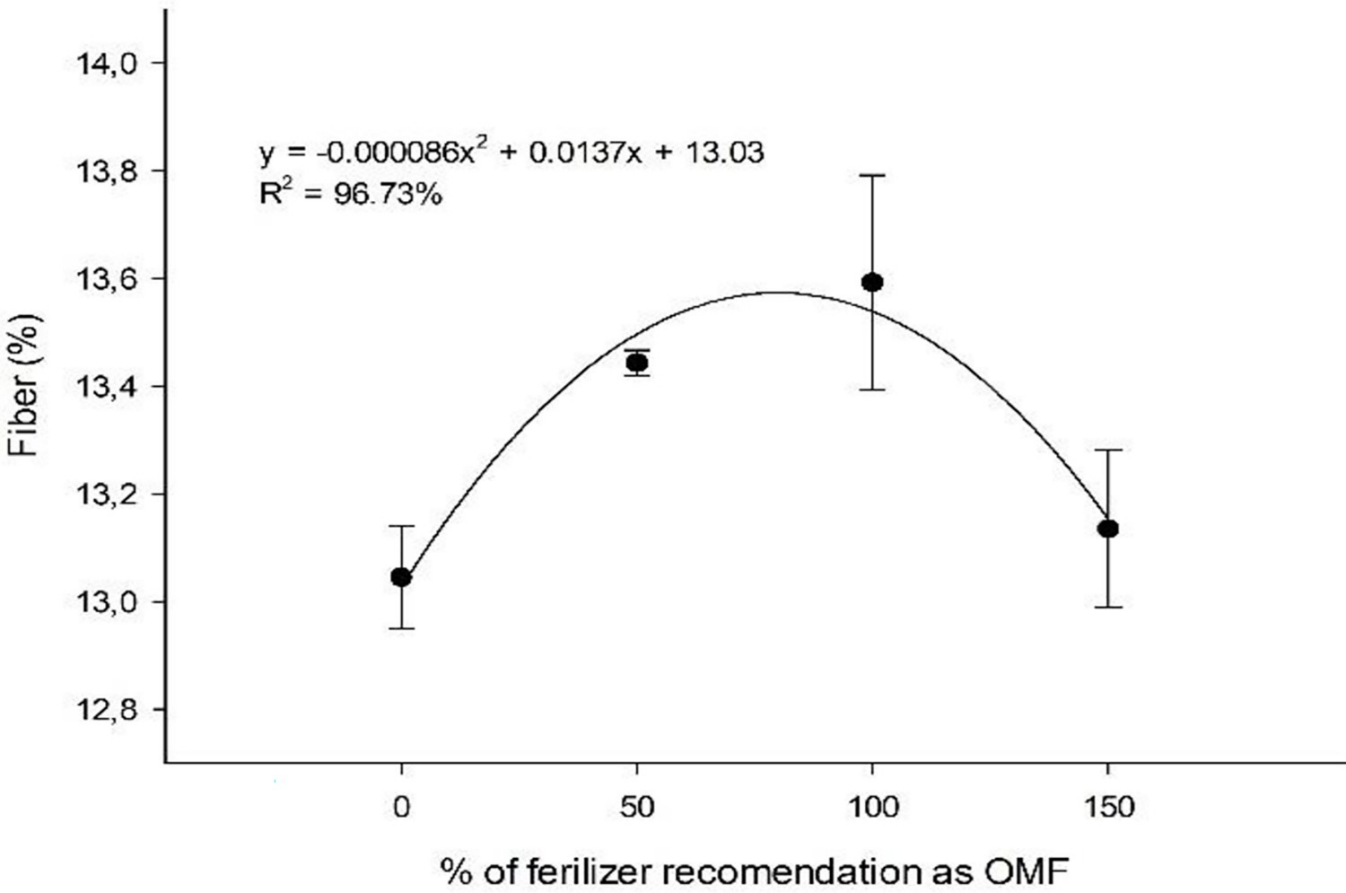
Sugarcane fiber content (%) under fertilizations with organomineral fertilizer based on sewage sludge at the first cut.

At the first cut, the highest fiber contents were associated with sewage sludge source (13.6%), which was 79.7% of the recommended dose (Fig 8). In addition, there were no significant interaction effects of application rate and OM source on fiber (%) at the second cut, with an average fiber content of 12.86%.

**Fig 9:**
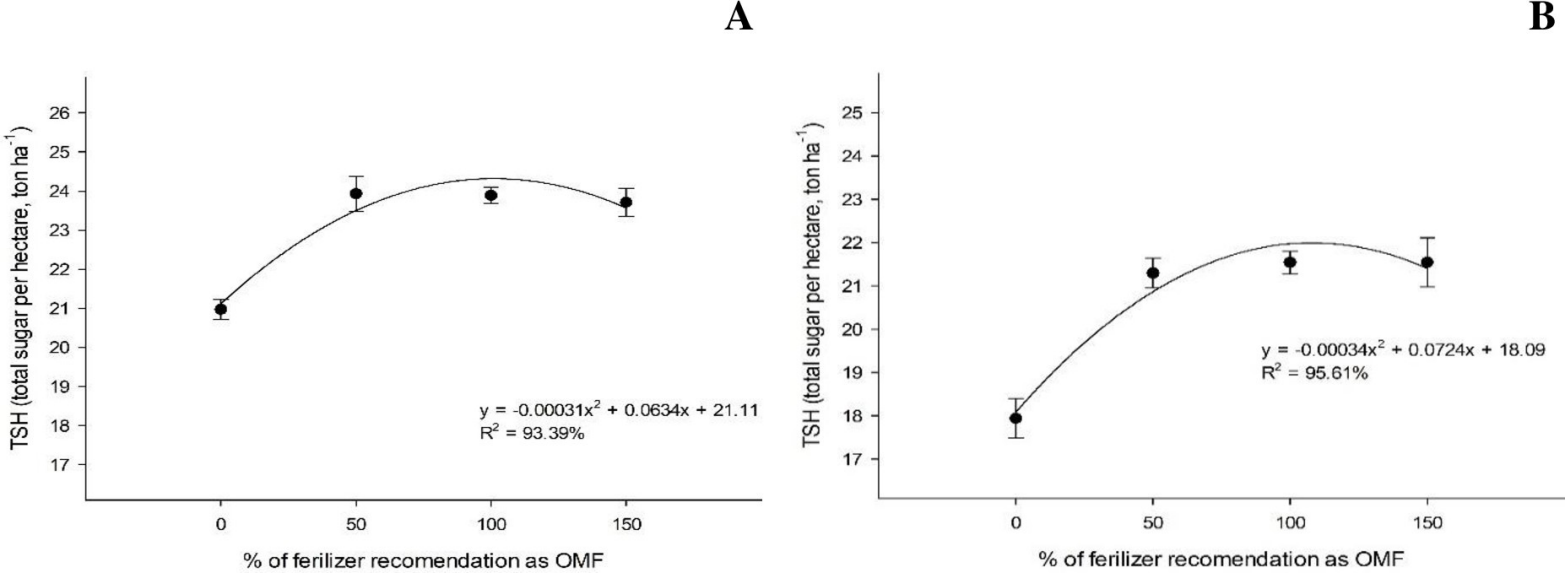
Total sugar per hectare (TSH, ton ha^-1^) under organomineral fertilizer application based on sewage sludge at the first (A) and second cut (B).

The highest sugar production per hectare observed at the first and second cut were 24.3 ton ha^-1^ and 21.9 ton ha^-1^, respectively, under 102.3 and 106.5% organomineral application rates, respectively, using either sewage sludge or sugarcane filter cake as organic sources (Fig 9).

Joint analyses of first and second cut yields revealed that the first harvest always achieved average yields higher than or equal to the second harvest, but never lower (Tables 6 and 7).

**Table 6:** Joint analyses of sugarcane sucrose contents, soluble solid contents, purity, and fiber content between the first (366 days after planting) and second (376 days after the first harvest) sugarcane harvests.

Treat.	Pol		Brix		Purity		Fiber	
	First	Second	First	Second	First	Second	First	Second
NC	13.88 A^1^	12.91 B	19.15 A	17.76 B	87.70 A	87.12 A	13.05 A	12.89 A
SS50	15.35 A	14.39 B	20.68 A	19.19 B	89.50 A	89.70 A	13.19 A	12.79 A
SS100	15.33 A	14.60 A	20.79 A	19.43 B	89.50 A	90.22 A	13.59 A	13.02 A
SS150	15.33 A	14.21 B	20.81 A	18.92 B	88.69 A	90.35 A	13.14 A	13.06 A
NC	13.88 A	12.91 B	19.15 A	17.76 B	87.70 A	87.12 A	13.05 A	12.89 A
FC50	14.87 A	14.44 A	20.48 A	19.19 B	88.61 A	89.81 A	13.90 A	12.64 B
FC100	15.22 A	13.63 B	20.61 A	18.40 B	89.02 A	88.79 A	13.19 A	12.90 A
FC150	15.04 A	14.10 B	20.59 A	18.69 B	88.77 A	90.14 A	13.63 A	12.71 B
Control	15.03 A	14.16 B	20.51 A	18.94 B	88.98 A	90.54 A	13.59 A	13.44 A
LSD	0.73		0.84		1.73		0.78	
CV (%)	3.54		3.01		1.36		4.18	
S.W.	0.10	0.09	0.11	0.10	0.11	0.08	0.14	0.09
L.	0.56	0.26	2.24	0.54	1.47	0.52	1.41	2.10

Treat.: fertilization treatments applied to sugarcane crops. SS: organomineral fertilizer based on sewage sludge; FC: organomineral fertilizer based on sugarcane filter cake; NC: negative control; Pol: sucrose content in sugarcane syrup; Brix: soluble solid contents in sugarcane syrup; Purity: Pol/Brix×100 (%); Fiber: water-insoluble biomass content.

^1^Averages followed by the same capital letter in a row indicate similar results between the sugarcane cuts based on Tukey’s test (p < 0.05). LSD: least significant difference. S.W.: Shapiro-Wilk test for normality (p > 0.01: normal distribution). L.: Levene’s test for homogeneity of variance (p > 0.01: all are homogeneous).

**Table 7:** Joint-analyses of sugarcane crop yield variables between the first (366 days after planting) and second (376 days after the first harvest) sugarcane harvest.

Treat.	StH		TSH		Prod	
	First	Second	First	Second	First	Second
NC	72.66 A^1^	53.86 B	20.97 A	17.95 B	151.12 A	139.06 A
SS50	74.11 A	65.59 A	23.65 A	21.41 B	154.05 A	148.86 A
SS100	100.30 A	90.75 A	24.37 A	24.00 A	159.12 A	163.98 A
SS150	83.34 A	81.70 A	23.45 A	22.26 A	152.99 A	156.71 A
NC	72.66 A	53.86 B	20.97 A	17.95 B	151.12 A	139.06 A
FC50	79.15 A	80.36 A	24.21 A	21.19 B	162.80 A	146.98 B
FC100	83.05 A	78.08 A	23.41 A	20.48 B	153.87 A	150.26 A
FC150	81.65 A	75.86 A	23.97 A	20.84 B	159.36 A	147.85 B
Control	76.29 A	74.61 A	23.90 A	21.55 B	158.91 A	152.01 A
LSD	14.40		1.49		7.32	
CV (%)	13.22		4.77		3.36	
S.W.	0.15	0.13	0.11	0.09	0.10	0.15
L.	1.38	0.69	0.66	0.64	1.42	2.25

Treat.: fertilization treatments applied to sugarcane crops. SS: organomineral fertilizer based on sewage sludge. FC: organomineral fertilizer based on sugarcane filter cake. NC: negative control. StH: estimated stalk per hectare (ton ha^-1^). TSH: total sugar produced per hectare (ton ha^-1^) Prod: Sugarcane biomass productivity (ton ha^-1^) TRS: total recovered sugar (kg ton^-1^).

^1^Averages followed by the same capital letter in a row indicate similar results between the sugarcane cuts based on Tukey’s test (p < 0.05). LSD: least significant difference. S.W.: Shapiro-Wilk test for normality (p > 0.01: normal distribution). L.: Levene’s test for homogeneity of variance (p > 0.01: all are homogeneous).

## Discussion

Generally, there were no differences in sugarcane yield properties following fertilization exclusively via mineral fertilizer or via organomineral fertilizers, indicating that organomineral fertilizers are a viable alternative for adoption in sugarcane crop production. The alternatives proposed in the present study could minimize the adverse impacts of successive application of mineral fertilizer, which are associated with the loss of soil biodiversity and long-term dependence on external inputs [49, 50]. In addition, considering the pre-treatment requirements for each source of OM, the organomineral fertilizers applied in the present study are safe and suitable for adoption in sugarcane production activities.

The organic fraction in organomineral fertilizers is an important aspect of these fertilizers. The fraction could positively alter nutrient cycling dynamics in the soil–plant system [51]. Several studies have reported organomineral fertilizers as viable substitutes for mineral fertilizers in many crops [15, 52–56]. Furthermore, long-term field demonstrated reductions in soil stocks of C and N nutrients in the deep soil layers following the application of large quantities of synthetic mineral fertilizers, which was not observed under organomineral fertilizers [57]. Organomineral fertilizers also reduce nitrogen (NH_3_-N) volatilization compared to exclusive mineral fertilizers [58].

Sugarcane filter cake also has considerable P fractions, an element essential for agriculture in the Cerrado because of its low natural availability in the soils of the biome [59]. In addition to providing essential nutrients, organomineral fertilizers potentially enhance soil chemical quality because of an increase in the soil negative charges, which increases the cation exchange capacity (CEC), and reduces the concentrations of exchangeable aluminum (Al^3+^) [60–62]. Soil OM is correlated with regular applications of N and P, and high soil CEC was observed when sewage sludge was used as the source of OM for an organomineral fertilizer [63]. The monitoring the environmental conditions and the orientation of farmers on how to manage this raw material (sewage sludge) is also essential to avoid heavy metal pollution and biological contamination during the reuse and management of this environmental liability [63].

Organomineral fertilizers based on treated sewage sludge can efficiently replace mineral fertilizer in maize [64] and sorghum [65] production activities, while improving soil fertility even at reduced doses, and without heavy metal pollution. In soybean, reduced doses (25% less compared to mineral fertilizer) of pelletized organomineral fertilizer formulated with treated sewage sludge or sugarcane filter cake increased plant height and stem diameter [16, 66]. The level of P present in sewage sludge compost depends on the chemicals used in the residue treatment process to improve P concentrations [67]. In addition, organomineral fertilizers release nutrients into the environment gradually, improving fertilizer efficiency [68]; therefore, the P derived from organic residue is a complementary strategy of supplying labile P to weathered soils [69].

The N:P ratio in organomineral fertilizers based on sewage sludge as the organic source is typically greater than that required by plants. In soils that receive sewage sludge-based organominerals, plants accumulate more P than plants grown exclusively with mineral fertilizers. P accumulation rates in plants are influenced by interactions between climate and soil factors, especially factors that affect the relationship between P adsorption and desorption in soil [70]. In addition, P release rate in soil is influenced by soil microbiota diversity and composition [11, 71, 72], and by the residual effects of the organomineral fertilizers based on sugarcane filter cake, which can last for up to four years following application [73, 74].

Other authors [75, 76] have concluded that the application of organic residues, such as cattle manure, replenishes organic acids responsible for restricting the adsorption of P by continuously blocking the sites of nutrient adsorption. Such a blocking effect is influenced by the P concentrations in organic residue. P concentrations below 0.2% of the total P indicate that P immobilization in the soil solution is greater than organic P mineralization.

The soil P concentrations in a maize study [54] increased from 6 mg kg^-1^ (without fertilizer) to 24 mg kg^-1^ following the use of mineral fertilizer and to 56 mg kg^-1^ following the use of organomineral fertilizer. The results of the maize study are similar to those observed in the present study. Sugarcane production with organomineral fertilizer formulated with filter cake was comparable to the production under mineral fertilizer. Root performance is also correlated with the effect of P on plant metabolism, with the presence of P being fundamental for good rooting and sugarcane tillering, with direct impacts on final yield and sugar production [15]. In addition, increments of P enhance sucrose contents, initial plant development, and P leaf content [77, 78]. In addition, in wheat, filter cake application increases grain yield and interrupts yield gain at doses greater than 60 ton ha^-1^ [79].

The organic fraction present in treated sewage sludge and sugarcane filter cake organominerals enhances soil aggregation, density, porosity, aeration, infiltration, and water retention capacity [80]. Such factors are essential in sugarcane cultivation in months with low rainfall and under cultivation with no irrigation [56, 81]. In eucalyptus, sewage sludge compost increased plant dry matter, with a gain of 50% compared to that observed under mineral fertilizer, indicating that this source of OM can replace mineral fertilizer [82]. Organomineral fertilizers also have the potential to reduce reliance on mineral fertilizer imports. The reduction of costs associated with P importation, for example, also improves crop productivity due to improved energy and water use [83], increasing the competitiveness of the agricultural produce in the market. For example, the P in sewage waste can satisfy 15–20% of the world’s demand supplied by phosphate rock [84].

Supplementation of the organic source with mineral elements can improve the responses from both resources (organic and mineral fractions), with lower environmental impact and higher agronomic efficiency. Greater sugarcane yield was achieved in a 15 t ha^-1^ of filter cake and 350 kg ha^-1^ of NPK fertilizer combination [85, 86]. In a study on the effects of organic fertilizers based on different organic sources (chicken bed, sugarcane filter cake, sugarcane vinasse) in sugarcane in comparison with basic NPK fertilizer, the highest numbers of roots were associated with the use of sugarcane filter cake [80]. This great root system is positively correlated with the high productivity observed in the first and second cuts. Conversely, in the present study, no substantial increase in production was observed between the first and second cuts [56]. However, the maintenance or even increase in stalk productivity was observed in 13 out of 25 clones, with increase productivity following organomineral fertilizer application [87].

The values observed for Brix in the present study were higher than 18% for both harvests. The optimal Brix was 18%. Brix has a direct relationship with sugar content and corresponds to 18 to 25% of the total sugar [88]. Sucrose content (pol, %) is also an important index in the sugar industry and is expected to be above 14% [89]. Sucrose contents below 14% were observed in the treatment without fertilizer (negative control) at the first cut.

Sugarcane juice purity reflects the potential of the juice to produce sugar. In the present study, the organomineral fertilizer (sewage sludge or filter cake) treatments yielded results similar to that of the mineral fertilizer, and were all above 85% at both harvests. The value exceeds the ideal value estimated by the sugar industry [89], and millers in the industry could reject shipments with a purity < 75% [42]. Similarly, fiber content (12–13%) of all treatments at the first cut was within the ideal range considered adequate (10–13%) [89, 90], highlighting how organominerals could provide adequate amounts of nutrients to sugarcane crops.

Positive sugarcane responses have been observed at 120% organomineral fertilizer application rates [91], which is similar to the observation in the present study. In addition, consistent with the findings of the present study, adverse effects on sugarcane qualitative parameters are minor or rare following fertilization with organomineral fertilizer [12, 92, 93], highlighting organomineral fertilizers as favorable alternatives in sugarcane cultivation. Both the first and second harvests after sugarcane planting presented trends expected for sugarcane crops, where the first cut is usually the most productive [94] with the attributes of the first harvest being superior or similar to those of the second harvest.

Organomineral fertilizers formulated with sewage sludge and filter cake increased soil P, B, and Mn levels in a maize crop soil significantly in a previous study [22]. The authors also reported that even at the highest application rate, fertilizer based on sewage sludge did not increase the soil heavy metal concentrations. Such a result can be attributed to the low concentrations of heavy metals originally present in sewage sludge collected for use in fertilizer production, as the release of industrial waste released into sewage collection networks is often prohibited. Despite the various potential benefits of the agricultural use of sewage sludge, the chemical composition of sludge should be monitored constantly because it can be a source of microbiological and chemical contamination to the soil. Nevertheless, some studies have reported that its use is generally safe and beneficial for agricultural crops [28].

Another study on the agronomic effects of the application of an industrial biosolid for maize crops verified that Cd and Pd contents were maintained within acceptable limits even at high application rates and with repeated applications [95]. Conversely, other authors [96] analyzed maize plant tissues and reported that Cd, Cr, Ni, and Pb had levels below the limits of detection. The use of sewage sludge in a mixture with concentrated mineral fertilizers to formulate organomineral fertilizer offers opportunities for their application over extensive areas and the use of equipment generally used for the application of regular mineral fertilizers to apply them in the field.

According to the results of the present study, organomineral fertilizers could replace mineral fertilizers in sugarcane cropping systems worldwide without negatively affecting critical sugarcane quantity and quality attributes. OM source type (sewage sludge or sugarcane filter cake) had little influence on the finding; however, proper care must be exercised to collect, manage, and treat any OM sources appropriate. That way, a renewable resource, whose improper disposal can have adverse environmental impacts, finds sustainable use in sugarcane production.

## Conclusions

The recommended organomineral fertilizer application rate to obtain optimal quantitative and qualitative sugarcane attributes was between 102 and 109% of the recommended mineral fertilizer dose (between 581.4 kg.ha^-1^ and 621.3 kg.ha^-1^ of organomineral fertilizer), regardless of the organic sources (filter cake or sewage sludge) in the first sugarcane harvest. In the second sugarcane harvest, the organomineral fertilizer based on treated sewage sludge increased the total amount of sugar per hectare and the quantity of sugarcane produced by 4.68 and 4.19%, respectively, when compared to the organomineral fertilizer based on sugarcane filter cake.

Organomineral fertilizers prepared using treated sewage sludge (with pollutants at acceptable levels) and sugarcane filter cake as sources of organic matter are viable alternatives to exclusive mineral fertilizers, with both economic and environmental benefits for sugarcane cultivation activities.

## Supporting information

S1 Data (XLSX)
